# Machined Surface Quality Monitoring Using a Wireless Sensory Tool Holder in the Machining Process

**DOI:** 10.3390/s19081847

**Published:** 2019-04-18

**Authors:** Zhiyuan Lu, Meiqing Wang, Wei Dai

**Affiliations:** 1 School of Mechanical Engineering and Automation, Beihang University, Beijing 100191, China; rselzy@buaa.edu.cn (Z.L.); wangmq@buaa.edu.cn (M.W.); 2 School of Reliability and Systems Engineering, Beihang University, Beijing 100191, China

**Keywords:** surface quality monitoring, wireless sensory tool holder, feature extraction, deep forest

## Abstract

The quality of a machined surface plays a critical role in assembly performance, especially for precise matching parts, and therefore it is necessary to develop a surface quality monitoring system in the machining process. In this paper, an indirect surface quality monitoring approach is proposed with a wireless sensory tool holder. First, experimentation is conducted to collect the machining process signals from the tool holder. Then, the time domain, frequency domain and time–frequency domain features are extracted, and the deep forest algorithm is adopted to identify the surface quality, which is evaluated through the surface average parameter. Finally, the results of the experiment and the comparisons with other approaches demonstrate the effectiveness of the proposed method, which could be applied to ensure the surface quality, improve the machining efficiency and reduce the rejection rate of the machining process.

## 1. Introduction

In mechanical manufacturing, surface quality is one of the key factors for the performance of products. Low surface quality will cause many defects, such as abnormal assembly, appearance, low fatigue strength and poor corrosion resistance, and affect service life [1]. Therefore, it is important to assure that the surface quality of mechanical products meets technical requirements. A large amount of research [2,3,4] has emphasized surface quality and many factors affecting the surface quality have been found, such as cutting parameters, tool wear and workpiece material. In industry, operators often select some recommended combination of cutting parameters according to some working reference or their experience to ensure the required surface quality and productivity. However, there are various factors that could affect the surface quality, which are still under-studied. For example, uneven materials or worn tools will lead to fluctuations in surface quality. To control the surface quality, setting cutting parameters cannot be relied upon alone. Therefore, the surface quality, mainly including surface roughness, waviness and errors of form, are usually measured offline after the machining process is already finished. If the machining quality does not meet requirements, the machining process should be stopped and adjusted in time. For some precision parts, the traditional approach is to apply manual finishing operations to achieve requirements, which is a time-consuming process. Thus, it is crucial to develop a surface quality monitoring system to control the quality online instead of offline.

The monitoring methods of surface quality are classified into direct and indirect methods. Direct monitoring methods, such as the laser method, ultrasonic method and fringe field capacitive method, can be applied to monitor the surface roughness directly [5,6,7]. The direct methods are vulnerable to field conditions, and they require a special device which is difficult to install or use. In indirect monitoring methods, some sensor signals corresponding to machining, including vibration, cutting force, sound, acoustic emission, temperature, spindle torque, spindle power and current, are widely used to monitor the surface quality. Although the monitoring accuracy of indirect methods is lower than that of the direct methods, they can be applied easily during the practical machining process.

Due to the rapid improvements in computing and Internet of Things technologies, a large amount of research into the monitoring and control of the surface quality based on sensor signals has been proposed and developed in recent years [8,9,10,11,12,13]. Luo et al. [14] proposed a surface quality monitoring approach, where real-time vibration signals were collected and analyzed by an adaptive spline wavelet algorithm, and the root sum square of the wavelet power spectrum was used to reflect the surface quality. Thomas et al. [2] analyzed the correlation between surface roughness and tool vibration, tool dynamic forces and cutting parameters for lathe dry turning processes. Quintana et al. [3] proposed a reliable surface roughness monitoring method for ball-end milling operations, where geometrical cutting factors, dynamic factors, part geometries lubricants, materials and machine tools were considered as the input of artificial neural networks. Khorasani [4] developed a general dynamic surface roughness monitoring system for milling operations by using the vibrations of a milling machine, and the cutting parameters, material type, coolant fluid were also considered. Plaza et al. [15] presented a surface roughness prediction approach for computerized numerical control (CNC) finish turning operations, where mechanical vibration signals were collected and singular spectrum analysis was utilized to decompose the vibration signals, and the signal characterization could be extracted to develop the roughness prediction model. Later, Plaza et al. [16] developed another surface roughness monitoring application for CNC turning operation by using vibration signals. In that paper, the wavelet packet transform was used to extract the related features of vibration signals to measure the surface roughness. Huang [17] presented an application for real-time surface roughness monitoring based on the intelligent neural-fuzzy model for end milling operations by using the cutting force and machining parameters. Agustina et al. [18] analyzed the method for evaluating the surface quality during robot-assisted polishing processes by using the acoustic emission signals, and the features calculated from the acoustic emission signals were obtained to monitor the surface quality.

Multiple sensors have also been extensively applied simultaneously to evaluate the surface quality. Axinte [19] presented research into estimating the surface quality by using multiple sensors, such as acoustic emission, vibration and cutting forces. In that paper, the time and frequency domain analysis of raw signals were utilized to evaluate the machined surface quality. Segreto et al. [20] proposed a surface roughness monitoring system for robot-assisted polishing by using several different sensor signals, such as acoustic emission, strain, and current signals, where two feature extraction methods—the statistics method and wavelet packet transform—were utilized to calculate the relevant features to construct the input feature vectors for a neural network to classify the surface quality level. Bhuiyan et al. [21] used the machine tool vibration and acoustic emission signals to monitor the surface roughness, where the fast Fourier transform was used to convert the raw signals and the key frequencies of vibration signals and acoustic emission signals were obtained. Barai et al. [22] developed a fuzzy inference system for the in-process estimation of surface roughness during the machining process, where the speed force component, radial force component, feed force component, vibration and acoustic emission signals were used as the input of the estimation model.

Although a great deal of research has emphasized online monitoring based on the data collected from the wired sensors mounted on the machine tool, this is costly and impractical for industrial application because of its long deployment time. The data transmission of wired sensors requires cabling, which hinders implementation in industry. To date, two methods could be utilized to address these issues. One is to use the numerical control (NC) kernel data to evaluate the surface quality [23,24,25,26,27]. The advantage of this method is that the data originating from the NC machine can be collected through only one Ethernet cable between the machine and PC, and the rest of the work can be achieved at the software level, which will not interfere with the operation of workers, greatly improving the efficiency and reducing the cost of data acquisition. However, the sampling frequency of NC kernel data is usually below 10Hz, resulting in a low response rate and poor monitoring performance, and many signal processing methods are not suitable for low-frequency data. In addition, only some advanced NC systems have the function modules to acquire the kernel data of machine tools, and the most common system in the literature is the NC of Siemens 840D, leading to this method being limited. The other method is to use wireless sensors, which have been applied widely in many fields such as health, environment, home and agriculture [28,29,30]. In the manufacturing industry, wireless sensors are utilized in industrial mobile robots, inventory management, equipment monitoring, and environment monitoring [31]. However, most wireless sensor are universal sensors, and few wireless sensors are designed for machining process monitoring. Therefore, few researchers have concentrated on wireless sensor research in surface quality monitoring. Recently, a wireless sensory tool holder (SPIKE) from Pro-Micron GmbH & Co. KG (Kaufbeuren, Germany) was developed, which could measure the torque forces, bending moments and temperature of the tool holder during the machining process. In this way, the data collected from the tool holder could directly reflect the current machining condition.

In this paper, the surface quality is monitored in real-time during the milling process based on the bending moment data collected from the wireless sensory tool holder. The experimentation is carried out to obtain the input and output data. Based on these data, the features can be extracted with the fast Fourier transform (FFT) and wavelet packet transform (WPT) statistical methods. The deep forest algorithm is adopted to monitor the surface quality in the milling process. In addition, the surface roughness average parameter Ra is adopted as an indicator of surface quality in the process of the application of the proposed method.

The rest of this article is organized as follows. The theoretical scheme and deep forest learning algorithm are proposed in Section 2. Section 3 describes the experimental setup. In Section 4, the results of the experiments and some discussions are presented. Finally, conclusions of this work and suggestions for further research are provided in Section 5.

## 2. Theoretical Scheme

**Figure 1:**
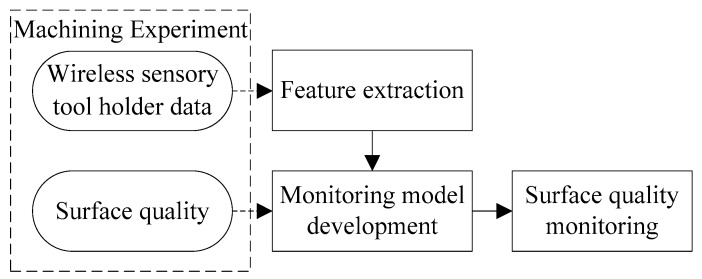
Flowchart of the proposed scheme.

The raw data collected from the wireless sensory tool holder is very large in volume. Therefore, the raw data should be processed to reduce the dimension, and the features of raw data should first be extracted in the time domain, frequency domain and time–frequency domain. Then, these features are used as the inputs for the monitoring model of surface quality, and the deep forest algorithm is utilized as the classifier. The flowchart of the proposed scheme is shown in Figure 1.

### 2.1. Features Extraction

For the statistical features in the time domain, some statistics including the absolute mean value, root mean square value, standard deviation, peak-to-peak value, peak factor, kurtosis, kurtosis factor, crest factor, pulse factor, form factor, amplitude of RMS, average amplitude, skewness, and skewness factor are calculated in this paper, and they are described in Table 1, where *x_i_* is the observation, *n* is the sample size and $\bar{x}$ is the mean of the sample.

The FFT and statistical methods are used to extract the frequency domain features, including the absolute mean value, root mean square value, standard deviation, peak-to-peak value, peak factor, kurtosis, kurtosis factor, crest factor, pulse factor, form factor, amplitude of RMS, average amplitude, and the skewness and skewness factor of the spectrum, which could reflect the characteristics of frequency.

The WPT and statistical methods are utilized to extract the time domain features at different frequency bands. The raw signals are decomposed by WPT, and some statistics including the absolute mean value, root mean square value, standard deviation, peak-to-peak value, peak factor, kurtosis, kurtosis factor, crest factor, pulse factor, form factor, amplitude of RMS, average amplitude, and the skewness and skewness factor of sensitive frequency bands are calculated.

### 2.2. Modeling for Surface Quality Monitoring

In the monitoring model of surface quality, many machine learning algorithms, such as artificial neural networks (ANNs), support vector machine (SVM), k-nearest neighbor (KNN), fuzzy logic, Bayesian networks, and decision trees, are widely applied in the classification of the design for surface quality level. For these algorithms, small-sample data can be used to train the model. However, it would take much time to study the prior properties of the data, and the classification accuracy remains to be improved. Deep learning, as a particular kind of machine learning, has recently been widely studied and applied. When the amount of data is large, the performance of a model trained by deep learning is much better than traditional machine learning algorithms. Although the deep learning algorithm has achieved great success in many fields, it is rarely applied in machining process monitoring due to the difficulty of acquiring a large amount of machining process data. In this work, the data acquisition using a wireless sensory tool holder is more convenient and feasible compared with wired sensors, and during normal machining tasks, a large amount of data can be obtained. Thus, in order to achieve better performance, the deep learning algorithm is used to train the surface quality monitoring model based on large amounts of machining process data.

The convolutional neural network [32] (CNN) is the most widely utilized deep learning approach and has achieved great success in image recognition, speech recognition and so on. However, it relies heavily on high-end hardware because of the large amount of computation, and many hyper-parameters need to be optimized. The deep forest is an ensemble approach based on decision trees, which has highly competitive performance to CNN in a large range of tasks [33]. This algorithm requires fewer parameters, and it is easy to train the model. In addition, for theoretical analysis, deep forest should be easier to handle than CNN. Therefore, the deep forest algorithm is used to train the monitoring model for surface quality in this paper.

The architecture of the deep forest can be divided into two structures. The first is multi-grained scanning, which fuses the multi-resolution characteristics to obtain the diverse representation of inputs. Another is the cascade forest structure, where the feature information calculated by each level of the cascade is processed for the next level, and each hidden layer is composed of several different types of random forests [34]. For the classification task, supposing that there are *h* categories, the input ***x*** ∈ *R^n^*, and *s* windows of different size representing *s* different resolution characteristics, the diverse information is then extracted under each resolution characteristic. For instance, there are *n*-dimensional raw feature vectors, the window size is *ws*, and the stride is 1; thus, a total of (*n* − *ws* + 1) feature vectors, which have *ws* features, can be generated as follows:

(1) $$x\in R^{n}\overset{Window=ws}{\underset{Stride=1}{\to }}{\left\{\tilde{x_{i}}\in R^{ws}\right\}}_{i=1}^{n-ws+1}$$

Each feature vector ${\tilde{x}}_{i}$ will be processed by a random forest, and the *h*-dimension outputs ***y_i_*** as follows:

(2) $${\tilde{x}}_{i}\in R^{ws}\overset{Forest}{\to }y_{i}\in R^{h}$$

Then, the outputs will be concatenated as transformed features as follows:

(3) $${\left\{y_{i}\in R^{h}\right\}}_{i=1}^{n-ws+1}\overset{Cascade}{\to }y=({\tilde{y}}_{1},\ldots ,{\tilde{y}}_{n-ws+1})\in R^{(n-ws+1)h}$$

In order to describe the diversity, two types of random forests are used including random forests and completely random tree forests. Finally, an *m*-dimensional transformed feature vector after multi-grained scanning will be generated based on the number of windows and random forests.

The transformed feature vector $y_{c}\in R^{m}$ is the input of the cascade forest structure, where *c* is the cascade of features under the different sliding windows. Suppose that there are *N* cascade layers, where two completely random tree forests and two random forests are used. The transformed features are embedded in each layer of the cascade forest network in a cascading manner to reserve the information of each layer. Thus, after the processing of *N* layers, the number of features remains unchanged, which is *m* + 4 × *h*. Finally, four random forests are used as the classifiers, and the prediction result can be obtained by averaging and maximizing. Detailed information about the deep forest are described in the literature [33].

## 3. Experimental Procedure and Data Collection

**Figure 2:**
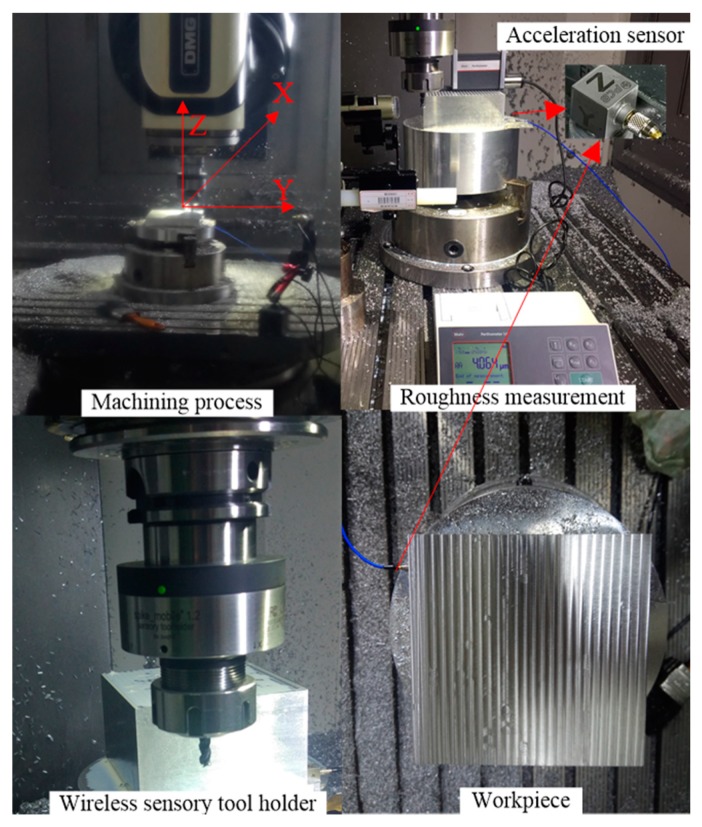
Experiment environment.

In this section, the proposed method for surface quality monitoring with a wireless sensory tool holder is evaluated experimentally. The real milling experiment was designed to collect the supporting data to train the surface quality monitoring model. The proposed approach runs on a server with a 2.40 GHz processor and 64 GB RAM. The machine tool is a five-axis DMG MORI with Numeric Control Siemens 840D sl. The machining material used for the experiments is aluminum alloy 7109. The cutting tool is a cemented carbide three-flute end milling cutter, and the diameter is 6 mm. A wireless sensory tool holder (Type: HSK63) manufactured by Pro-Micron GmbH & Co. KG was installed in the machine tool to replace the universal tool holder. The data collected from the tool holder were transmitted to the PC software through a receiver unit with one antenna wirelessly. The sampling rate of wireless sensor is 2500 Hz/channel. In order to illustrate the effectiveness of the proposed method based on the wireless sensory tool holder signals, vibration signals in three directions (*X*, *Y* and *Z*) during the milling experiment process were also collected by a wired triaxial acceleration sensor (Type: PCB 356A32) mounted on the machining material. NI-9234 was used for data acquisition. The sampling rate of the acceleration sensor is 51,200 Hz/channel. The experiment environment, the wireless sensor layout and the acceleration sensor layout are shown in Figure 2.

The machining operation was carried out with spindle speed of 6000 rpm, feed rate of 2000 mm/min, and a depth of cut of 1 mm in the z direction. A new cutting tool was used to cut the workpiece. With the exacerbation of tool wear, the surface quality became worse. In this experiment, the surface roughness average parameter Ra is adopted as the indicator of surface quality of the workpiece, which was measured by a surfagauge (Type: Mahr M1-set) after completing each surface cutting. The surface quality is divided into four levels according to Ra. The evaluation length was 17.50 mm and the basic length was 2.50 mm. The Ra was measured four times and the mean value was considered. The machining needs to be stopped for cooling after each cut because of the dry milling process. The analysis results are presented and discussed below.

## 4. Results and Discussion

### 4.1. Signal Preprocessing

In this work, the wireless sensory tool holder could collect the bending moment in *X* and *Y* directions, as well as the axial force, torque, and temperature during the machining process. In the milling process, the direction of the cutting force on the tool holder is constantly changing. Thus, the bending moment of the tool holder, which equals the product of the cutting force and the moment arm, is more suitable for analysis compared with the cutting force and can directly reflect the current machining condition. Therefore, the bending moment signals in *X* and *Y* directions are collected, and both components are vector additions to equal the total amount of bending moment, which is used for analysis and monitoring.

**Figure 3:**
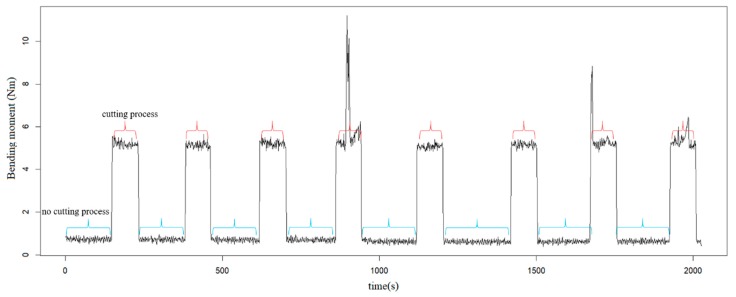
The bending moment signals.

During the milling process, there are a large number of spindle idling processes; thus the collected data can be separated into four situations: no cutting process, the entrance or exit of the cutter, and the cutting process. Figure 3 shows the raw bending moment signals for one surface cutting. In order to train the surface quality monitoring model with the collected real-time tool holder information, a trigger method should be developed to remove the invalid information, and only the cutting process information is collected for monitoring. The bending moment signals are used as the input for the trigger, and the output trigger returns 0 for no cutting process, 1 for entrance or exit of the cutter, and 2 for the cutting process. When the output is 2, the data during this period are collected and adopted for monitoring.

**Figure 4:**
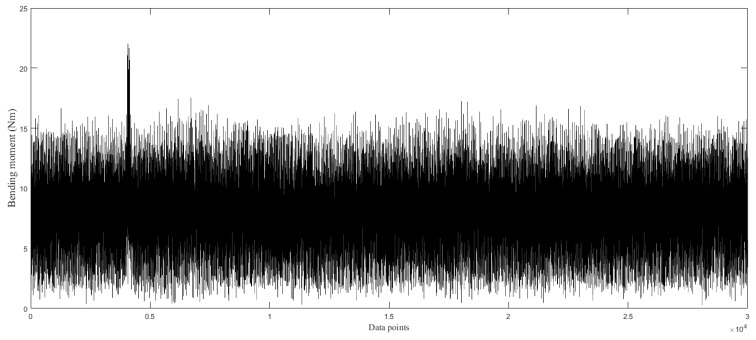
The bending moment signals during cutting process for each surface cutting.

Then, the bending moment signals during the cutting process for each surface cutting can be obtained, as shown in Figure 4.

**Figure 5:**
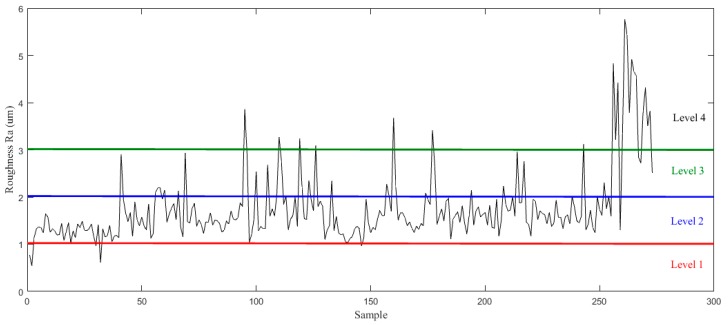
Roughness curve.

The surface roughness Ra was measured after each surface cutting, and the change curve of roughness is shown in Figure 5, where the surface quality is divided into 4 levels.

**Figure 6:**
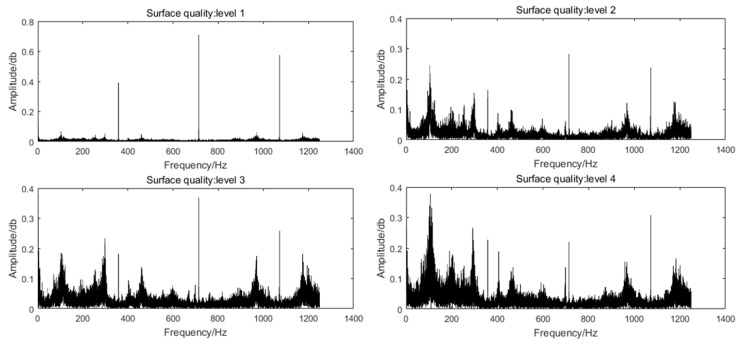
FFT of bending moment signals of different surface quality levels.

All bending moment signals are analyzed by FFT to obtain the sensitive frequencies of the cutting situation so that the sensitive frequencies can be selected for wavelet packet decomposition. The bending moment signals of different surface roughness levels are taken for FFT analysis, as shown in Figure 6. The results show that the frequencies related to the cutting situation are mainly concentrated around 100~150 Hz, 350~400 Hz, 700~750 Hz and 1050~1100 Hz. The sampling frequency is 2500 Hz, and so the second degree wavelet is utilized to reveal the details of the specialized frequency band.

**Figure 7:**
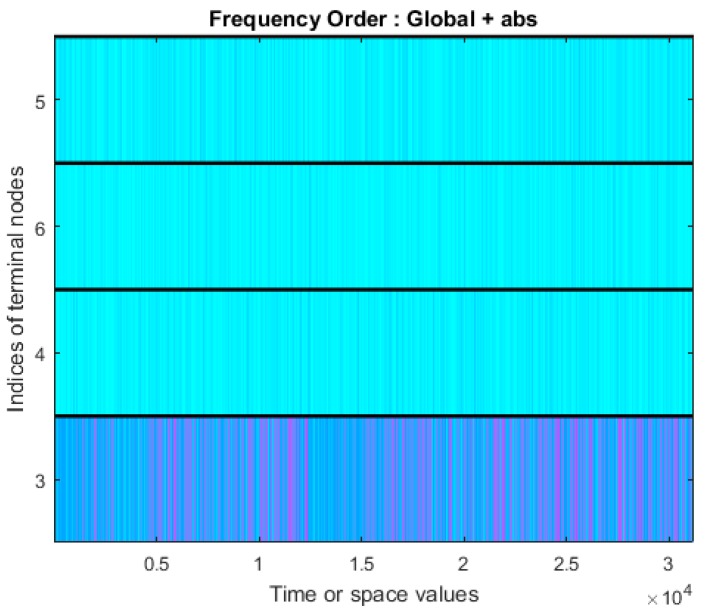
The wavelet packet spectrum of the bending moment for surface quality level 4.

The bending moment signals are decomposed into the approximation component and detailed component, which represent the low-frequency band and high-frequency band, respectively. Db5 is used as the mother wavelet. The wavelet packet spectrum of signals for surface quality level 4 is shown in Figure 7. The statistical features including the absolute mean value, root mean square value, standard deviation, peak-to-peak value, peak factor, kurtosis, kurtosis factor, crest factor, pulse factor, form factor, amplitude of RMS, average amplitude, skewness and skewness factor of each raw bending moment signal, the spectrum data after FFT, each approximation component, and each detailed component are calculated, and there are 84 = (14 + 14 × 4 + 14) features.

### 4.2. Modeling for Surface Quality Monitoring

Based on the extracted features, the features vectors for training the deep forest are constructed. In this work, 273 surfaces were cut; therefore, 273 surface roughness values were measured. In order to increase the training sample size and improve the monitoring efficiency, the collected data during the cutting of one surface is divided into multiple samples, which correspond to the one surface roughness. The data collected every 1 s is taken as one sample. All samples are randomly divided into two sets, training sets and validation sets, and validation sets account for 15% of the total samples. There are two sizes of data scanning windows for the multi-grained scanning of the deep forest—4 and 6—and the scanning stride is 1. The other parameters are set as follows: max layers: 100; min_samples_mgs (minimum sample size when training multi-grained scanning random forest): 20; min_samples_cascade (minimum sample size when training cascade random forest): 20; tolerance: 0. The accuracy of the deep forest model in the training sets reached 99.54% and in the validation sets reached 90.91%, and the average response time (feature extraction time plus classification time) was 0.9652 s.

### 4.3. Comparisons with Other Approaches

#### 4.3.1. Comparison with Other Algorithms

The same datasets were adopted to identify the surface quality with KNN, ANN and SVM. The monitoring performance used for these models is evaluated with the classification accuracy in training sets and validation sets, respectively, and the average monitoring response time, which were calculated based on all samples.

For training the KNN model, the number of nearest neighbors *K* and the distance of the vector space are the significant parameters, and the performance of the KNN model with the different parameter settings is summarized in Table 2, where AT is the accuracy in training sets, AV is the accuracy in validation sets, and ART is the average response time. It can be seen in Table 2 that the best accuracy in the training sets reached 82.68% and the best accuracy in the validation sets reached 80.95%, and the average response time was within 0.1 s.

For ANN, the two-layer feed-forward backpropagation network was trained. The transfer function of the hidden layer was ‘logsig’, and ‘purelin’ was the linear transfer function for the output layer, the learning rate was set as 0.1, and the training goal was 1 × 10^−10^. The other parameters and performance are summarized in Table 3. It can be seen in Table 3 that when the training function is Levenberg–Marquardt, the best accuracy in the training sets reached 100%, while the best accuracy in the validation sets only reached 76.19%; when the training function is gradient descent with an adaptive learning rate, the best accuracy in the training sets reached 91.85%, while the best accuracy in the validation sets only reached 77.50%. With the continuous iteration of the algorithm, the accuracy in the training sets improved, while the accuracy in the validation sets decreased due to overfitting. The average response time of ANN is around 0.1 s.

For training the SVM model, a multi-SVM was used as the classifier and the Gaussian kernel function was applied for SVM. The optimal penalty coefficient *C* and the kernel function coefficient *G* were obtained using the grid search algorithm in the library for support vector machines toolbox. The best *C* and *G* were 0.03125 and 0.03125, respectively. The accuracy in the training sets reached 78.35%, the best accuracy in the validation sets reached 76.19%, and the average response time was within 0.1 s.

According to the comparison with the experiment results, it can be seen that the deep forest has the best classification accuracy in the training sets and validation sets. Although the average monitoring speed of the deep forest is 0.9652 s, which is the slowest compared with other methods, it is timely enough for the monitoring of surface quality with the wireless sensory tool holder, because the sampling rate of the wireless sensor is 2500 Hz and the data collected every 1 s are taken as one sample in this work.

#### 4.3.2. Comparison with Wired Sensor

The vibration signals in three directions (X, Y and Z) from the acceleration sensor were analyzed, and the monitoring model was trained with the same scheme. The data collected every 1 s are taken as one sample. There are two sizes of data scanning windows for the multi-grained scanning of deep forest, 4 and 6, and the scanning stride is 1. The other parameters are set as follows: max layers: 100; min_samples_mgs (minimum sample size when training multi-grained scanning random forest): 10; min_samples_cascade (minimum sample size when training cascade random forest): 7; tolerance: 0. The accuracy of the model based on vibration signals in the training sets reached 98.53% and in the validation sets reached 87.50%. In addition, the average monitoring speed reached more than 3 s. In the machining process, the placement of the acceleration sensor is far from the machining area compared with the wireless sensory tool holder, making the vibration signals susceptible to interference from the manufacturing system, while the information from the wireless sensory tool holder could directly reflect the machining condition. In addition, the data volume of high-frequency vibration signals in three directions is quite large, leading to a slow monitoring speed. Therefore, the monitoring model based on the data from the wireless sensory tool holder performs better than the model based on vibration signals for surface quality monitoring in terms of accuracy and speed. In addition, the price of the wireless sensory tool holder is around 15,000 Euros, which is not expensive compared to some vibration sensors.

### 4.4. Discussion

Surface quality can be monitored by taking advantage of the wireless sensory tool holder and deep forest algorithm. When the proposed method is deployed in industrial practice, it should be noted that not all machining condition monitoring can apply this method due to the low sampling frequency of wireless sensors. Although the sampling frequency of wireless sensory data is much higher than that of NC kernel data, it is far from enough for some machining anomaly monitoring which requires a high response speed, such as tool breakage detection. Obviously, the wired sensor can acquire high-frequency feature information of this kind of anomaly because of the high sampling frequency and shorter response time. Therefore, the monitoring requirements need to be analyzed before selecting suitable sensors.

In addition, in this paper, each training sample has its own surface quality label, and the deep forest algorithm has excellent monitoring performance for this kind of data. However, the deep forest algorithm relies too heavily on labels, and it is inadequate for unsupervised learning. If the machining process changes, the monitoring performance of the deep forest model would be affected. Thus, the deep forest model in this paper can be utilized in a relatively stable machining process. For a complicated multistage machining process, some unsupervised learning algorithms are more suitable. Furthermore, the computation speed of the deep forest is relatively slow compared with common algorithms, such as ANN, SVM and KNN. When a high response speed is required but accuracy is less important, these algorithms may be more appropriate.

In summary, the proposed approach is quite effective in surface quality monitoring. In contrast to other approaches based on wired sensors for surface quality monitoring, the proposed approach has two advantages: firstly, the information collected from the wireless sensory tool holder can directly reflect the machining condition, which results in excellent monitoring performance; secondly, the placement of a sensory tool holder is quite convenient for any worker and it can continuously monitor the machining surface quality (up to 16 h) and will not interfere with the operation of workers. The application of this paper is a milling process, but the proposed approach can be applied to other machining process such as drilling, grinding, turning, threading, reaming, rolling and friction stir welding. Besides the monitoring of surface quality, this approach can also be used for other machining condition monitoring tasks that do not require an extremely high response speed in industrial practice, such as the detection of tool wear and chatter.

## 5. Conclusions and Future Work

In this work, a surface quality monitoring approach is presented based on the wireless sensory tool holder. The wireless sensory tool holder is adopted to collect the bending moment signals during the milling process. The raw signals are analyzed by different methods including statistical methods, FFT and WPT, and the different features of the raw signals are extracted in the time domain, frequency domain and time–frequency domain. The deep forest algorithm is utilized to identify the different surface quality levels, which are evaluated based on the surface roughness Ra parameter. The accuracy of the monitoring model in the training sets reached 99.54%, and it reached 90.91% in the validation sets, which outperforms the other methods in our comparison. In this way, the proposed method can be applied in industrial practice to monitor surface quality and provide a reference for operators to ensure surface quality, improve machining efficiency and reduce the rejection rate of the machining process.

The major limitation at the moment is that the application of the proposed method requires information about corresponding machining parameters, workpiece materials and cutting tools. Different machining processes need different monitoring models. An adaptive monitoring system is currently being considered. Besides, the monitoring indicator of surface quality is not only the surface roughness, but also the surface waviness, surface texture, surface scratch, and some mechanical properties of the surface layer, and these indicators should be considered simultaneously in future work. Furthermore, the information from the wireless sensory tool holder could be applied to detect some other machining anomalies such as tool wear and chatter, which will also be taken into account. All of the above works will help to extend the practicability of the proposed method and wireless sensors in industrial practice.

## Figures and Tables

**Table 1:** Statistical features.

No.	Feature	Symbol	Equation
1	Absolute mean value	*X_u_*	$X_{u}=\frac{1}{n}\sum {}_{i=1}^{n}\left|x_{i}\right|$
2	Root mean square value (RMS)	*X_rms_*	$X_{rms}=\sqrt{\frac{1}{n}\sum {}_{i=1}^{n}x_{i}{}^{2}}$
3	Standard deviation	*X_std_*	$X_{std}=\sqrt{\frac{\sum {}_{i=1}^{n}(x_{i}-\bar{x})}{n-1}}$
4	Peak-to-peak value	*X_p_*	$X_{p}=\max(x_{i})-\min(x_{i})$
5	Peak factor	*X_pk_*_f_	$X_{pkf}=\frac{\max(\max(x_{i}),\;-\min(x_{i}))}{X_{rms}}$
6	Kurtosis	*X_k_*	$X_{k}=\frac{\sum {}_{i=1}^{n}{\left(x_{i}-\bar{x}\right)}^{4}/n}{{\left(\sum {}_{i=1}^{n}{\left(x_{i}-\bar{x}\right)}^{2}/n\right)}^{2}}$
7	Kurtosis factor	*X_kf_*	$X_{kf}=\frac{X_{k}}{X_{rms}^{4}}$
8	Crest factor	*X_cf_*	$X_{cf}=\frac{\max(\max(x_{i}),\;-\min(x_{i}))}{X_{ar}}$
9	Pulse factor	*X_psf_*	$X_{psf}=\frac{\max(\max(x_{i}),\;-\min(x_{i}))}{X_{u}}$
10	Form factor	*X_ff_*	$X_{ff}=\frac{X_{rms}}{X_{u}}$
11	Amplitude of RMS	*X_ar_*	$X_{ar}={\left(\frac{1}{n}\sum {}_{i=1}^{n}\sqrt{\left|x_{i}\right|}\right)}^{2}$
12	Average amplitude	*X_aa_*	$X_{aa}=\frac{1}{n}\sum {}_{i=1}^{n}\left|x_{i}\right|$
13	Skewness	*X_s_*	$X_{s}=\frac{\sum {}_{i=1}^{n}{\left(x_{i}-\bar{x}\right)}^{3}/n}{{\left(\sqrt{\left(\sum {}_{i=1}^{n}{\left(x_{i}-\bar{x}\right)}^{2}/n\right)}\right)}^{3}}$
14	Skewness factor	*X_sf_*	$X_{sf}=\frac{X_{s}}{X_{rms}^{4}}$

**Table 2:** The parameter settings and performance of k-nearest neighbor (KNN). AT, accuracy in training sets; AV, accuracy in validation sets; ART, average response time.

Number of Nearest Neighbor	Distance: ‘Euclidean’			Distance: ‘Cosine’			Distance: ‘Correlation’		
	AT (%)	AV (%)	ART (s)	AT (%)	AV (%)	ART (s)	AT (%)	AV (%)	ART (s)
*K* = 5	80.95	73.81	0.0913	82.25	80.95	0.0884	82.25	80.95	0.0993
*K* = 10	80.52	73.81	0.0831	82.68	78.57	0.0936	82.68	78.57	0.0789
*K* = 15	78.35	76.19	0.0957	82.25	80.95	0.0987	82.25	80.95	0.0934
*K* = 20	78.35	76.19	0.0932	82.25	80.95	0.0903	82.25	80.95	0.0965
*K* = 25	78.35	76.19	0.0758	82.25	80.95	0.0852	82.25	80.95	0.0833
*K* = 30	78.35	76.19	0.0843	78.35	76.19	0.0933	78.35	76.19	0.0856

**Table 3:** The parameter settings and performance of the artificial neural network (ANN).

**Iterations**	**Training Function: Levenberg–Marquardt**								
	**Hidden Layer Nodes: 5**			**Hidden Layer Nodes: 10**			**Hidden Layer Nodes: 15**		
	**AT (%)**	**AV (%)**	**ART (s)**	**AT (%)**	**AV (%)**	**ART (s)**	**AT (%)**	**AV (%)**	**ART (s)**
100	97.85	67.50	0.1123	99.14	72.50	0.1284	100	65.00	0.0937
500	97.85	67.50	0.1263	99.14	57.50	0.0947	99.57	65.00	0.1222
1000	100	57.50	0.0987	100	65.00	0.1287	100	75.00	0.1534
5000	97.00	65.00	0.1324	100	76.19	0.1103	100	60.00	0.1785
10,000	98.28	57.50	0.1227	100	60.00	0.1352	100	52.50	0.1875
**Iterations**	**Training function: Gradient Descent with Adaptive Learning Rate**								
	**Hidden Layer Nodes: 5**			**Hidden Layer Nodes: 10**			**Hidden Layer Nodes: 15**		
	**AT (%)**	**AV (%)**	**ART (s)**	**AT (%)**	**AV (%)**	**ART (s)**	**AT (%)**	**AV (%)**	**ART (s)**
100	80.26	65.00	0.1287	80.26	65.00	0.1423	80.26	65.00	0.1554
500	84.12	75.00	0.1109	85.41	75.00	0.1299	84.98	77.50	0.1643
1000	85.41	75.00	0.1648	85.41	75.00	0.1756	85.41	75.00	0.1123
5000	86.27	75.00	0.0934	85.84	75.00	0.1913	86.70	75.00	0.1394
10,000	88.41	72.50	0.1433	88.84	72.50.	0.1656	86.27	75.00	0.0986
50,000	90.99	70.00	0.1022	91.42	72.50.	0.9923	91.85	67.04	0.1039
